# A Putative P-Type ATPase Regulates the Secretion of Hydrolytic Enzymes, Phospholipid Transport, Morphogenesis, and Pathogenesis in *Phytophthora capsici*

**DOI:** 10.3389/fpls.2022.852500

**Published:** 2022-05-10

**Authors:** Chengdong Yang, Bowen Zheng, Rongbo Wang, Hongyang Chang, Peiqing Liu, Benjin Li, Justice Norvienyeku, Qinghe Chen

**Affiliations:** ^1^Key Laboratory of Green Prevention and Control of Tropical Plant Diseases and Pests, Ministry of Education, College of Plant Protection, Hainan University, Haikou, China; ^2^Hainan Yazhou Bay Seed Laboratory, Sanya Nanfan Research Institute of Hainan University, Sanya, China; ^3^Fujian Key Laboratory for Monitoring and Integrated Management of Crop Pests, Institute of Plant Protection, Fujian Academy of Agricultural Sciences, Fuzhou, China

**Keywords:** *Phytophthora capsici*, PcAPT1, phospholipid transport, hydrolytic enzyme, pathogenesis

## Abstract

*Phytophthora capsici* is an important plant pathogenic oomycete with multiple hosts. The P4-ATPases, aminophospholipid translocases (APTs), play essential roles in the growth and pathogenesis of fungal pathogens. However, the function of P4-ATPase in *P. capsici* remains unclear. This study identified and characterized PcApt1, a P4-ATPase Drs2 homolog, in *P. capsici.* Deletion of *PcAPT1* by CRISPR/Cas9 knock-out strategy impaired hyphal growth, extracellular laccase activity. Cytological analyses have shown that PcApt1 participates in phosphatidylserine (PS) transport across the plasma membrane. Also, we showed that targeted deletion of *PcAPT1* triggered a significant reduction in the virulence of *P. capsici*. Secretome analyses have demonstrated that secretion of hydrolytic enzymes decreased considerably in the *PcAPT1* gene deletion strains compared to the wild-type. Overall, our results showed that PcApt1 plays a pivotal role in promoting morphological development, phospholipid transport, secretion of hydrolytic enzymes, and the pathogenicity of the polycyclic phytopathogenic oomycete *P. capsici*. This study underscores the need for comprehensive evaluation of subsequent members of the P-type ATPase family to provide enhanced insights into the dynamic contributions to the pathogenesis of *P. capsici* and their possible deployment in the formulation of effective control strategies.

## Introduction

The oomycetes are ubiquitous, filamentous eukaryotic organisms. They include numerous plant pathogens that pose a significant threat to global food security and natural ecosystems (Kamoun et al., 2015). Although oomycetes are morphologically similar to filamentous fungi, they belong to a distinct taxonomic group and falls kingdom Protista along with diatoms and brown algae (Tyler, 2001). Species within the oomycete subclass are highly diverse and are causal agents of devastating infectious diseases in various microorganisms, including plants and animals. For instance, the outbreak of the famous late blight of potato disease caused by *Phytophthora infestans*, the sudden oak death by *Phytophthora ramorum*, as well as root and stem rot disease in soybean caused by *Phytophthora sojae* are among some of the intensively studied microbial infections caused by pathogenic oomycetes (Judelson and Blanco, 2005; Tyler, 2007; Grünwald et al., 2012). *Phytophthora capsici*, on the other hand, accounts for substantial production losses in the wide variety of crops, including members in the family Solanaceae (tomato, pepper, eggplant, etc.), Cucurbits (cucumber, pumpkin, squash, cantaloupe, watermelon, etc.), and Leguminosae (snap bean, faba beans, etc.) (Lamour et al., 2012; Kamoun et al., 2015). Recent estimates showed that plant pathogenic oomycetes cause over one billion dollars losses worldwide in the vegetable production industry (Lamour et al., 2012). Although *P. capsici* is a pathogen of great economic importance, the molecular mechanisms of pathogenicity are not well understood.

Plants and pathogens are engaged in a dynamic co-evolutionary struggle for survival. Over time, plants have evolved a complex and versatile immune system to ward off potential pathogens and manage potentially beneficial microbes. Therefore, to successfully infect plants, plant pathogenic oomycetes deploy large arsenals of secreted effector proteins that act as weapons to promote invasion and colonization of (amino-terminal motif Arg-x-Leu-Arg, x represents any amino acid) and CRN (crinkling- and necrosis-inducing proteins) effectors. The archetypal oomycete cytoplasmic effectors are the secreted and host-translocated RxLR proteins. All oomycete avirulence genes (encoding products recognized by plant hosts and contribute to host defense response) identified so far encode RxLR effectors. CRN cytoplasmic effectors found in *P. infestans* transcripts encoding putative secreted peptides can elicit necrosis *in planta*, a characteristic of plant defense response. In recent decades, many secreted effectors and their targets have been identified in *Phytophthora* pathogens, and *Phytophthora* species perturb various host innate immunity using these secreted effectors, indicating that the deployment of a large arsenal of secreted effectors is an important aspect of plant pathogens pathogenicity (Whisson et al., 2007; Haas et al., 2009; Wang and Jiao, 2019).

P-type ATPases constitute a family of integral proteins that utilize the energy from ATP hydrolysis to the transport of ions and lipids across cell membranes (Axelsen and Palmgren, 1998; Palmgren and Nissen, 2011). Based on phylogenetic analysis, P-type ATPases have been organized into five main classes (P1-P5 ATPases) (Axelsen and Palmgren, 1998). P4-ATPases (aminophospholipid translocases, APTs/flippases of the type IV or Drs2 family) are unique in that they transport or flip phospholipids across membranes and are only found in eukaryotes. APTs maintain the asymmetrical distribution of aminophospholipids in membranes by translocating phosphatidylserine (PS) and/or phosphatidylethanolamine (PE) from one leaflet of the bilayer to the other. Phospholipid asymmetry is critical for membrane fusion events (vesicle budding and docking) at the plasma membrane and the trans-Golgi network (Hu and Kronstad, 2010). P4-ATPase worked on endocytosis and exocytosis by facilitating transport of phospholipids (such as phosphatidylserine) to maintain the asymmetry of phospholipids in the plasma membrane and endocrine membrane (Lopez-Marques et al., 2014). At present, the research on P4-ATPase is mainly concentrated in mammals, plants and yeast. In animal cells, P4-ATPase can actively flip phospholipids from the cytoplasmic lobules of the cell membrane to the cytoplasmic lobules. It plays an essential role in cell division, endocytosis and exocytosis, apoptosis, blood coagulation, and nerve growth, etc. (Coleman et al., 2013; Andersen et al., 2016). In plants, the silencing of *GbPATP* will cause cotton to be more sensitive to low temperatures (Liu et al., 2015). ALA1 enhances chilling tolerance in Arabidopsis (Gomes et al., 2000). In yeast, Drs2p and Dnf1p are involved in the process of endocytosis and exocytosis, and these proteins can maintain intracellular homeostasis (Liu et al., 2007).

In the context of phospholipid trafficking, some aminophospholipid translocases within the P-type ATPases are known to play roles in fungal growth and virulence. In *Magnaporthe grisea*, two flippases, MoPde1 and MoApt2, were essential for pathogenicity, and the aminophospholipid translocase MgApt2 is found to be crucial for exocytosis during plant infection by *M. grisea* (Balhadere and Talbot, 2001; Gilbert et al., 2006). In *Aspergillus nidulans*, the flippase AnDnfD is important for conidiation, and that AnDnfA and AnDnfB play complementary role in vegetative growth and PS asymmetry (Schultzhaus et al., 2015; Schultzhaus et al., 2019). In the opportunistic fungal pathogen *Cryptococcus neoformans*, the flippase Apt1 contributes to the stress response, polysaccharide export, and virulence (Hu and Kronstad, 2010; Rizzo et al., 2014). In *Fusarium graminearum*, recent studies have revealed that the flippases are involved in vegetative growth, asexual and sexual reproduction, and pathogenesis. Moreover, individual flippase plays distinct roles in regulating of DON biosynthesis in *F. graminearum* (Li et al., 2019; Zhang et al., 2019; Yun et al., 2020). However, the functions of flippases in vegetative growth, sporulation, and virulence of oomycetes, including *P. capsici*, remain unknown. In this study, we identified the *PcAPT1* gene, which encodes a putative aminophospholipid translocase and is functionally related to Drs2, P4-ATPase in *Saccharomyces cerevisiae*. We generated *PcAPT1* gene deletion strains for *P. capsici* using the CRISPR/Cas9 system. Our results indicate that the P4-ATPase gene *PcAPT1* plays an important role in the growth, laccase activity, stress resistance, pathogenicity, and the secretion of hydrolytic enzymes of *P. capsici*. This study provides a certain theoretical basis for further exploring the pathogenic mechanism of *P. capsici* and formulating effective control strategies.

## Materials and Methods

### Bioinformatics Analysis

Genomic DNA and protein sequences of aminophospholipid translocases (APTs) orthologs in *P. capsici* (PcApt1, PHYCA_120336), *S. cerevisiae* (ScDnf1, YER166W; ScDnf2, YDR093W; ScDnf3, YMR162C; ScDrs2, YAL026C), *N. crassa* (NcApt1, NCU06281; NcApt2, NCU03592; NcApt3, NCU00352; NcApt4, NCU07443; NcApt5, NCU03818), *A. nidulans* (AnDnfA, An8672; AnDnfB, An6112; AnDnfC, An2011; AnDnfD, An6614), *M. grisea* (MgPde1, AY026257; MgApt2, MGG_02767; MgApt3, MGG_04066; MgApt4, MGG_04852), and *F. graminearum* (FgDnfA, FGSG_08595; FgDnfB, FGSG_06743; FgDnfC1, FGSG_09020; FgDnfC2, FGSG_00595; FgDnfD, FGSG_05149) and were obtained from the online website.^1^ The phylogenetic relationship analysis of APTs in different species were conducted in MEGA7 by Maximum Likelihood method (with setting of 1000 bootstrap replications). The functional domains of APT orthologs in different species were predicted using online website.^2^

### Strains, Plants, and Culture Conditions

The *P. capsici* wild-type strain (LT1534) and the mutants generated in this study were incubated on 10% V8 agar media in a 25°C incubator with darkness. Vegetative growth assays of the tested strains were assayed on 10% V8 agar media in a 25^°^C incubator for 5-days. To promote sporangial production and zoospore release, the individual strains were cultured in 10% V8 agar media with darkness for 3-days, then under light for another 2-days. Sporangium produced by the indicated strains were washed with sterile distilled water and incubated at low temperature (12^°^C) for 0.5 and 2 h, respectively, for zoospore release. Ten microliter sporangium suspension was sampled to observed the release rate of zoospores under a light microscope. Stress response assays of the tested strains were conducted on 10% V8 agar media supplemented with or without 6 mM H_2_O_2_, 0.2M CaCl_2_, 0.4M NaCl, and 200 μg/mL CFW (Calcofluor White). Bell peppers were grown in a 25°C greenhouse under a 12-h light/12-h dark cycle before inoculation. Before inoculation, the etiolated hypocotyls of Bell pepper were grown in a 25^°^C greenhouse under dark conditions.

### Plasmid Construction and Generation of the *PcAPT1*-Knock-Out Mutants

All primers used in this study are listed in Supplementary Table 1. The sgRNA primers of *PcAPT1* were designed using the online website EuPaGDT^3^ and annealed as previously described (Fang et al., 2017). The sgRNA fragments targeting the *PcAPT1* gene were then cloned into the single all-in-one plasmid (pYF515) with *Nhe*I and *Bsa*I digestion, which has the Cas9 and sgRNA cassettes. The eGFP fragment and ∼1 kb homologous flanking sequences of the *PcAPT1* gene were amplified and linked to the linearized pBluescript II KS + to obtain gene replacement constructs plasmid using ClonExpress Ultra One Step Cloning Kit (Vazyme). The *PcAPT1* gene knock-out mutants were generated by the CRISPR/Cas9-mediated gene replacement strategy according to previous description (Fang et al., 2017). The complemented strain of the Δ*Pcapt1* mutant was generated as described previously (Zhang et al., 2021). In brief, full-length *PcAPT1* was amplified using related primer pairs (Supplementary Table 1) and then inserted into the plasmid pTOR with *Eco*RI and *Cla*I digestion. The resulting pTOR-*PcAPT1* construct was sequenced in the company (BioSune, Shanghai, China) to identify its successful insertion into the plasmid. The recombinant plasmid was introduced into the Δ*Pcapt1* protoplast.

### Quantitative Real Time PCR

Strains involved in this experiment were incubated in liquid 10% V8 media at 25^°^C for 2 days. Mycelia were then gathered from which total RNA was extracted and used to performed reverse transcription (RT) using the RT kit (Takara, RR047A) to generate cDNA to quantify transcription levels with TB Green kit (Takara, RR420A) using specific primer pairs (Supplementary Table 1) with β-tubulin as endogenous reference gene. The data generated was finally analyzed with 2^–ΔΔCT^ method as previously described (Livak and Schmittgen, 2001). Statistical analyses were performed by multiple *t*-tests from biological repeats using GraphPad Prism at *p* ≤ 0.01.

### Microscopic Observation

The indicated strains were incubated in liquid 10% V8 media for 48 h. For phospholipid observation, mycelia from the tested strains were stained with NBD-PS/PC/PE (Avanti Polar Lipids) following procedures described by Hanson and Nichols (2001). In brief, fresh mycelia were transferred to ice-cold MM-S media (0.5 g KCl/L, 0.5 g MgSO_4_⋅7H_2_O/L, 1.5 g KH_2_PO_4_/L, 0.5% Biotin, 2% sorbitol, pH6.5) with 10 μmol of lipid dye and incubated at 30°C with darkness for 5 and 30 min, then washed three times with cold MM-S media and observed under an Olympus BX51 microscope.

### Pathogenicity Assays

The infection ability of the tested strains on plants were determined by inoculating the detached leaves and etiolated hypocotyls of Bell pepper. Mycelial plugs (5 mm in diameter). At the same time, zoospore suspensions (100 zoospores per microliter) from the individual strains were used to independently inoculate detached leaves of Bell pepper. The inoculated leaves were incubated under high (75%) humidity and dark conditions at 25°C before examination for typical symptoms. Photographs were taken after 2-days post-inoculation (dpi). Statistical differences were calculated by multiple *t*-tests from three biological replicates using GraphPad Prism at *p* ≤ 0.05.

### Extracellular Enzyme Activity Assays

Detection of laccase secretion in the experimental strains were performed according to the previous procedure (Sheng et al., 2015). At least three biological replicates were conducted for these experiments.

### Extraction and Identification of Extracellular Proteins in the Culture Media From *Phytophthora capsici* (Wild-Type) and Δ*Pcapt1* Strains

The (wild-type) and Δ*Pcapt1* strains were cultured in synthetic liquid medium (Kamoun, 1993). The mycelia were first filtered out with 4-layers of cheese cloth. The resulting supernatants were further purified by filtering with a 0.22-mm Millipore membrane. (NH_4_)_2_SO_4_ was added to the clarified supernatant (spent media) in the ratio of (70 g:100 mL) while swirling the media. The content was incubated under 0°C overnight precipitated for the precipitation of total extracellular proteins. Collection, processing and identification of the precipitated proteins was performed according to methods described by Ma et al. (2015).

## Results

### PcApt1 Is a Member of P-Type ATPase Family

**FIGURE 1:**
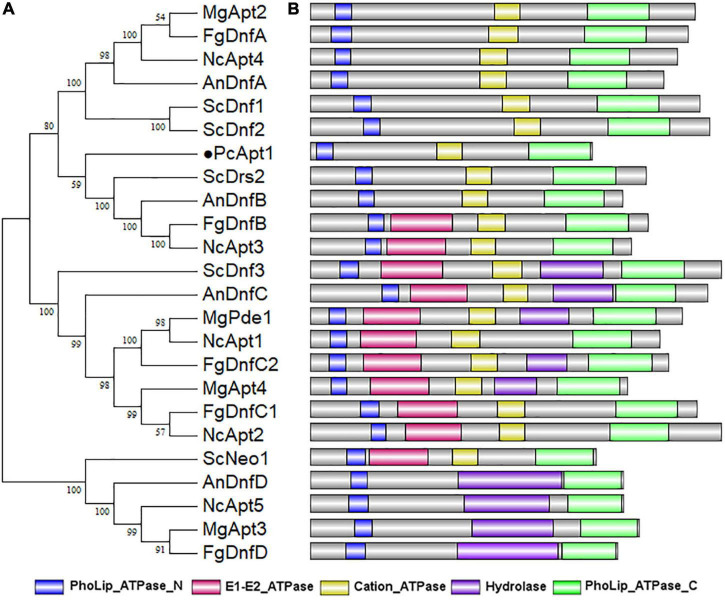
PcApt1 encodes a P-type ATPase. **(A)** The phylogenetic relationship analysis of APTs in *P. capsici*, *S. cerevisiae*, *N. crassa*, *A. nidulans*, *M. oryzae*, and *F. graminearum* were conducted in MEGA7 by Maximum Likelihood method with setting of 1000 bootstrap replications. **(B)** The functional domains of APT orthologs in different species were predicted using an online website (http://pfam.xfam.org/search).

To identify genes coding for aminophospholipid translocases (APTs) in *P. capsici*, we retrieved amino acid sequences of five genes encoding for aminophospholipid APTs (*Drs2*, *Dnf1*, *Dnf2*, *Dnf3*, and *Neo1*) in *Saccharomyces cerevisiae* (Hua et al., 2002) for BLASTp and reverse-BLASTp analyses. BLAST search identified *S. cerevisiae* APTs orthologs, including Drs2 referred to as PcApt1 (PHYCA_120336) in the *P. capsici* genome.^4^ Comparative phylogenetic analyses of Apt orthologs obtained from *S. cerevisiae*, *Neurospora crassa*, *Aspergillus nidulans*, *Magnaporthe grisea*, *Fusarium graminearum*, and *P. capsici* showed that PcApt1 and ScDrs2 shared a more recent common ancestor compared to Apt1/Drs2 identified in other fungi species (Figure 1A). Further functional domain search analyses revealed conserved functional domains motifs, including PhoLip_ATPase_N (Phospholipid-translocating P-type ATPase N-terminal), Cation_ATPase (Cation transport ATPase), PhoLip_ATPase_C (Phospholipid-translocating P-type ATPase C-terminal) in Drs2 orthologs from the individual species (Figure 1B). These results partially confirm PcApt1 as Drs2 ortholog in *P. capsici*.

### PcApt1 Is Important for Vegetative Growth in *Phytophthora capsici*

**FIGURE 2:**
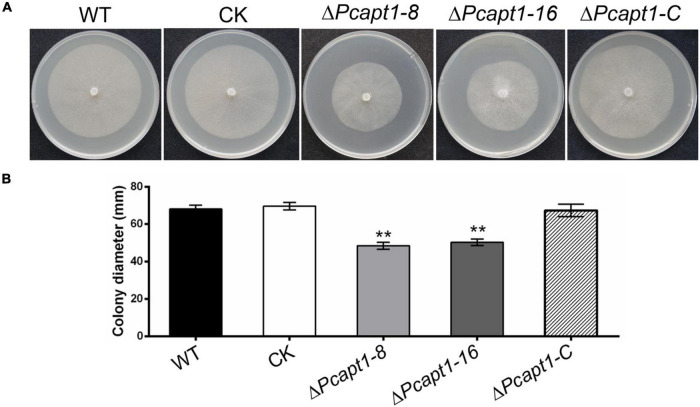
Deletion of *PcAPT1* reduce the vegetative growth of *P. capsici*. **(A)** The wild-type LT1534 (WT), complemented, CK, and the Δ*Pcapt1* mutant strains were cultured on 10%V8 agar media at 25°C and photographed at 5-days. **(B)** Average colony diameters of the experimental strains grown on 10%V8 agar media for 5-days. Statistical differences between the Δ*Pcapt1* mutant and three controls were calculated by multiple *t*-tests from three biological replicates using GraphPad Prism at **P ≤ 0.01.

To investigate the biological functions of the *PcAPT1* gene in *P. capsici*, we generated knockout mutants by using the CRISPR/Cas9-mediated gene replacement strategy (Fang et al., 2017). The mutants were validated using PCR and sequencing, which showed a 3.108 kb band with primer pairs F3/R3 in the *PcAPT1* mutants in contrast to a 5.799 kb band in the wild-type LT1534. Quantitative real transcription PCR (qRT-PCR) results further validated deletion of the *PcAPT1* gene in the Δ*Pcapt1* mutants (Supplementary Figure 1). Moreover, false positive transformants were used as negative control (CK). We next examined the vegetative growth of the wild-type LT1534, the Δ*Pcapt1* mutants, complemented and CK strains. After 5-days of incubating on 10% V8 agar media, the Δ*Pcapt1* strains showed slower growth. The growth rate was decreased by approximately 28% compared to the growth recorded for the wild-type LT1534 and CK strains (Figure 2). To generate the complemented (C1) strains, we re-introduced the pTOR-GFP cassette (Dai et al., 2021) containing the full-length PcAPT1 coding (ORF) sequence into the Δ*Pcapt1* knock-out mutant, qRT-PCR results have also demonstrated that the expression of *PcAPT1* gene in the complemented strain (Supplementary Figure 1). Vegetative growth of the complemented strain is comparable to WT and CK (Figure 2). These results showed that PcApt1 has crucial roles in the vegetative growth of *P. capsici*.

### PcApt1 Crucially Modulates Stress Tolerance in *Phytophthora capsici*

**FIGURE 3:**
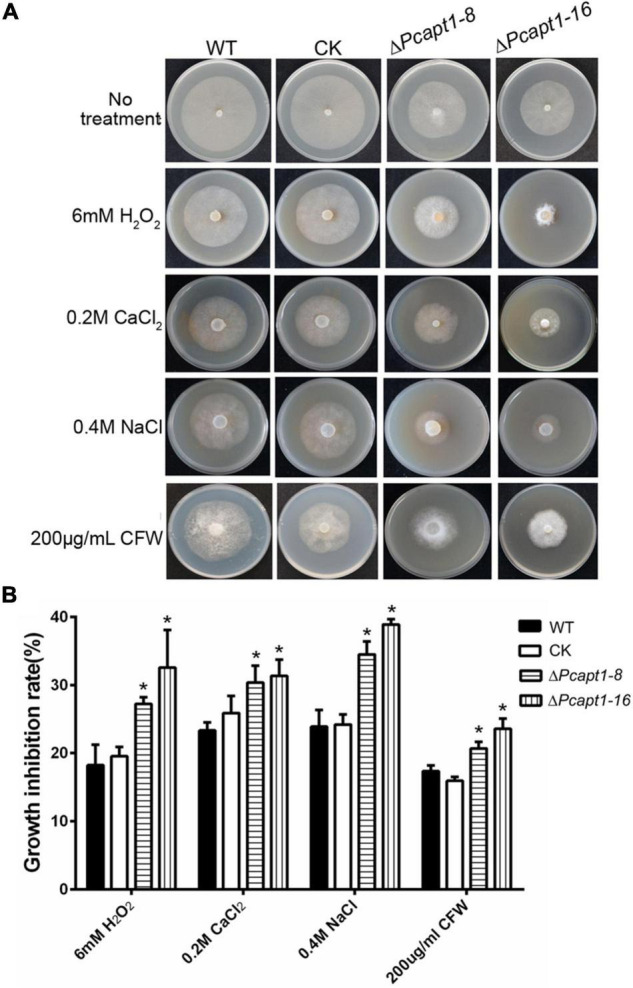
Environmental stress responses of the Δ*Pcapt1* mutants in *P. capsici*. **(A)** Colonies of the indicated strains incubating on 10%V8 agar media containing 6 mM H_2_O_2_, 0.2M CaCl_2_, 0.4M Nacl, and 200 μg/mL CFW (Calcofluor White) in a 25^°^C incubator for 5 days. **(B)** The inhibition in the growth of the individual strains cultured on 10%V8 agar media containing stress-inducing agents. Statistical differences were calculated by multiple *t*-tests with three independent replicates using GraphPad Prism at *P ≤ 0.05.

Phosphatidylserine is an essential component of the biological membrane and is also involved in sensing environmental signals (Hankins et al., 2015; Schultzhaus et al., 2015). Studies have shown that flippases play a key role in phosphatidylserine asymmetry distribution. In the pathogenic fungi *Cryptococcus neoformans*, the deletion of *APT1* compromised the tolerance of the defective strains to multiple stress conditions, including oxidative and nitrosative stress (Hu and Kronstad, 2010). To understand the role of PcApt1 in cell membrane-associated stress response in *P. capsici*. We assessed the growth of the individual strains on culture media supplemented with multiple stress-inducing osmolytes, including (hydrogen peroxide/H_2_O_2_, sodium chloride/NaCl, calcium chloride/CaCl_2_, and calcofluor white/CFW). Our results indicated that the Δ*Pcapt1* strains showed decreased tolerance, particularly H_2_O_2_, NaCl, and CaCl_2_, compared to the wild-type and CK strains (Figure 3). These results indicated that PcApt1 plays a critical role in modulating stress tolerance in *P. capsici*.

### PcApt1 Plays a Critical Role in the Pathogenicity of *Phytophthora capsici*

**FIGURE 4:**
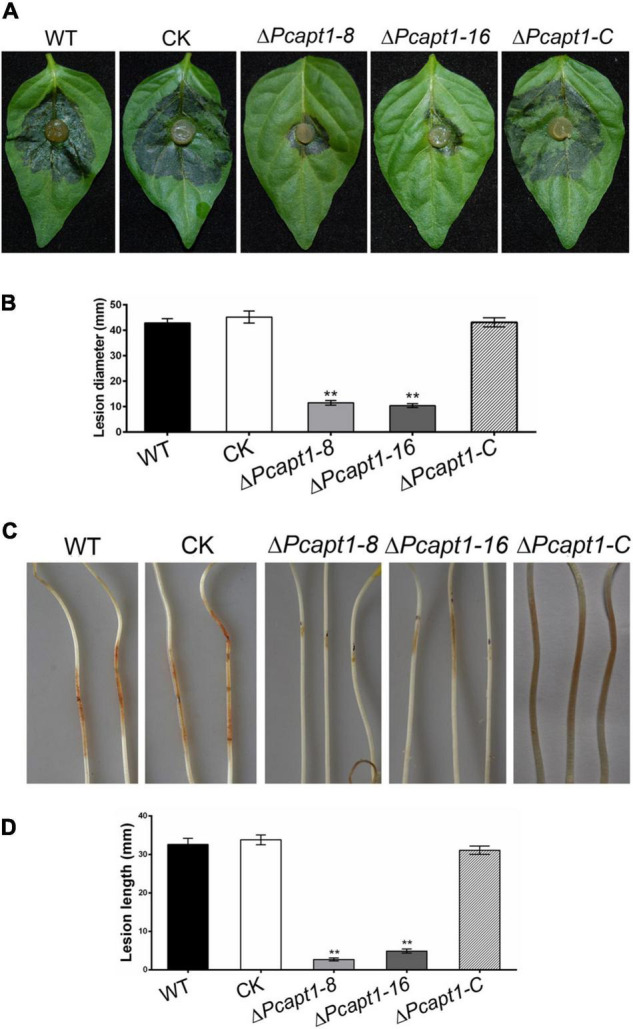
PcApt1 is required for virulence. **(A)** Infection of the Δ*Pcapt1* strains to detached Bell pepper leaves was significantly decreased. Bell pepper leaves were inoculated with mycelia plugs of WT, complemented, CK, and the Δ*Pcapt1* strains. Photographs were taken at 2 days post-inoculation (dpi). **(B)** Graphical representation of the lesion length in panel **(A)**. Lesion length on the Bell pepper leaves was measured after 2 days of inoculation. Statistical differences were calculated by multiple *t*-tests from three biological replicates using GraphPad Prism at **P ≤ 0.01. **(C,D)** Pathogenicity test of the indicated strains on hypocotyls of etiolated Bell pepper seedlings. Etiolated hypocotyls of Bell pepper were inoculated with mycelia plugs of WT, complemented, CK, and the Δ*Pcapt1* strains. Images were taken and lesion length were measured at 2 dpi. Statistical differences were calculated by multiple *t*-tests with three independent replicates using GraphPad Prism at **P ≤ 0.01.

To gain insight into the roles of PcApt1 in *P. capsici* pathogenicity, mycelium plugs of the wild-type LT1534, the Δ*Pcapt1* strains, complemented, and CK strains were used to inoculated the detached Bell pepper leaves. After 2-days post-inoculation (dpi) under high humidity at 25°C, and examined the symptoms. We observed that wild-type, complemented, and CK strains induced severe disease symptoms with an average lesion diameter of 43 mm on Bell pepper leaves. However, the Δ*Pcapt1* strains showed reduced virulence and induced minor (smaller) lesions with an average diameter measuring 11 mm on leaves of Bell pepper leaves (Figures 4A,B). The inoculation of etiolated hypocotyls of Bell pepper seedlings with the individual strains yielded similar virulence characteristics (Figures 4C,D). Moreover, inoculation of zoospores from the tested strains showed the similar results on the leaves (Supplementary Figure 2). These findings collectively revealed that PcApt1 significantly promotes the full virulence of *P. capsici*.

### PcApt1 Plays Essential Role in the Activities of Extracellular Laccases

**FIGURE 5:**
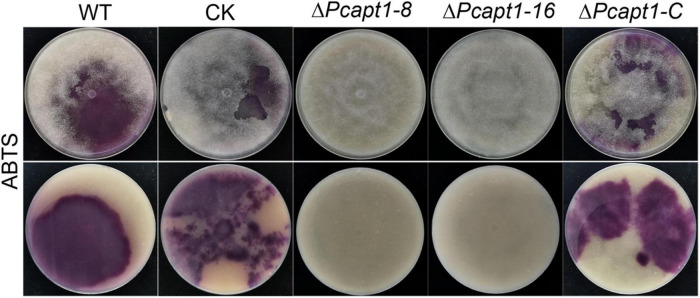
PcApt1 is critical for extracellular laccase secretion in *P. capsici*. Laccase activity assay. The individual strains were cultured on lima bean agar (LBA) media supplemented with 0.2 mM ABTS. Laccase activity of these strains was determined by monitoring oxidized ABTS (dark purple) in LBA media. The individual strains were photographed at 10-days post-incubation (dpi).

Studies have shown that laccases are a critical virulence factor in fungi (Zhu and Williamson, 2004; Chi et al., 2009). Therefore, we examined laccase activity by assessing the oxidation of ABTS (2, 2-azino-bis (3-ethylbenzothiazoline-6)-sulfonic acid). We observed that ABTS was readily oxidized (invisible) in the Δ*Pcapt1* strains; hence, it failed to produce dark-purple marks were formed around the mycelial mat. However, there was a visible manifestation of dark purple staining marks around the mycelia of the wild-type and the negative control strains indicating the non-oxidized state of ABTS (Figure 5). We inferred that PcApt1 essentially regulates the extracellular laccases activities to likely facilitate pathophysiological development of *P. capsici*.

### PcApt1 Participates in Phosphatidylserine Transport in *Phytophthora capsici*

**FIGURE 6:**
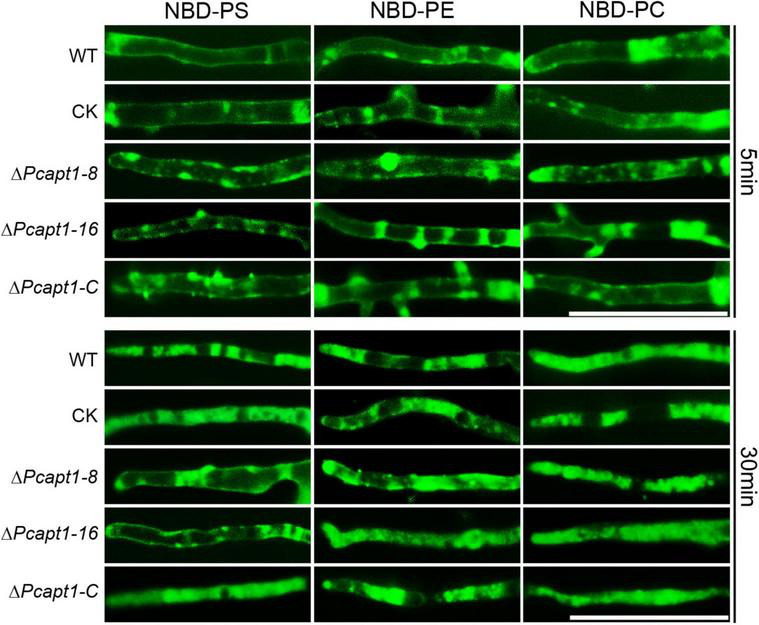
PcApt1 participates in phosphatidylserine transport in *P. capsici*. Fresh hypha from the tested strains were stained by NBD-PS/PC/PE in MM-S media with darkness for 5 and 30 min, then washed three times with cold MM-S media and observed under an Olympus BX51 microscope. Bar = 50 μm.

Studies on individual P4-ATPase family members from animals, plants and fungi have demonstrated that P4-ATPases have different substrate specificities. The substrate specificity characteristics of flippases are readily detectable with fluorescent phospholipids (Lopez-Marques et al., 2014). Accordingly, we visualized the cellular location of 7-nitro-2-1,3-benzoxadiazol-4-yl (NBD)-tagged phospholipids in the mycelia of the wild-type and Δ*Pcapt1* strains under a fluorescence microscope. These results revealed visible fluorescence signals in the plasma membrane and cytoplasm of mycelia obtained from the tested strains treated with NBD-phosphatidylethanolamine (NBD-PE), phosphatidylcholine (NBD-PC) and phosphatidylserine (NBD-PS) staining at 5 min. Moreover, the fluorescence signals labeled by NBD-PE and NBD-PC were mainly transferred to cytoplasm in the wild-type, Δ*Pcapt1*, complemented, and the CK strains after 30 min staining. By contrast, NBD-PS fluorescence signals were still remained on the plasma membrane but not totally transported into cytoplasm in the Δ*Pcapt1* strain until 30 min staining (Figure 6). Collectively, these results revealed that the PcApt1 likely contributes to phosphatidylserine transport in *P. capsici*.

### PcApt1 Regulates the Export of Hydrolytic Enzymes and Other Pathogenesis-Related Proteins in *Phytophthora capsici*

**FIGURE 7:**
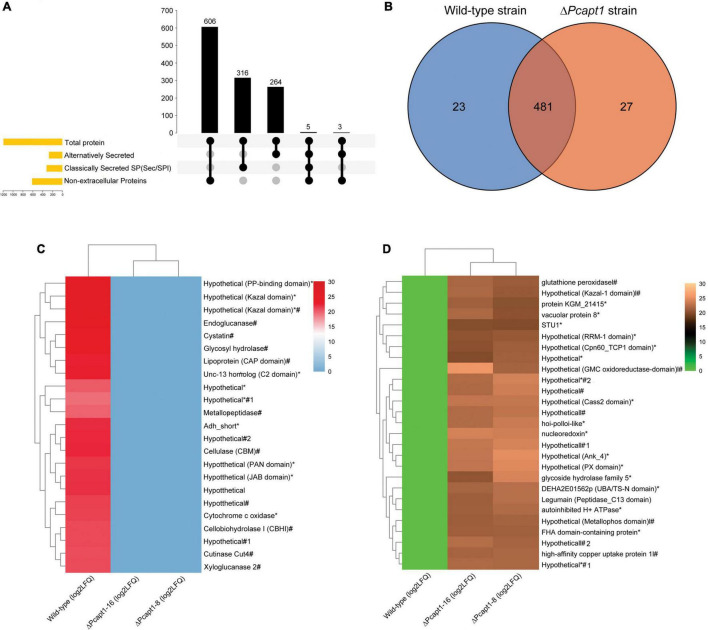
Predicted localization pattern, comparative distribution, and intensities of proteins recovered from the extracellular milieu of the wild-type and Δ*Pcapt1* strains. **(A)** The Upset-Venn portrays the localization pattern of total proteins present in the extracellulome data obtained for the wild-type and Δ*Pcapt1* strains. **(B)** The Venn graph showed the comparative distribution of extracellular proteins recovered from the extracellular milieu of the wild-type and Δ*Pcapt1* strains. **(C)** The heatmap represent the relative intensities of classical and non-classically secreted protein exclusively present in the extracellular milieu of the wild-type strain. **(D)** The heatmap represent the relative intensities of classical and non-classically secreted protein exclusively present in the extracellular milieu of the Δ*Pcapt1* strain. (*) attachments in the heatmap denote predicted non-classically secreted proteins, harsh (#) attachments in the heatmap denote predicted classically secreted proteins, while, (*#) denote proteins that are secreted through both classical and non-classical secretion pathways. Intensities were computed as [Log_2_ (average LFQ)] for the individual strains.

Besides facilitating the exit of secretory proteins from the ER, P4-ATPases also promotes the conventional and non-conventional transport and secretion of proteins and ions through the regulation of membrane curvature and the formation of intracellular transport vesicles (Schmitt, 2002; Maxwell et al., 2008; Qu and Dubyak, 2009). Here, we examined the impact of *PcAPT1* gene deletion on the accumulation of classical and non-classical secreted proteins in the extracellular milieu by subjecting spent liquid synthetic media obtained from culturing the individual strains after culturing. Total proteins present in the extracellular milieu of the respective strains were coagulated with (NH_4_)_2_SO_4_ and used for label-free protein extraction according to methods described by Ma et al. (2015). Results obtained from profiling of total proteins present in the extracellular milieu of Δ*Pcapt1* and the wild-type strains showed that while a total of 1,220 proteins were identified, 1,142/1,220 proteins quantifiable (Supplementary Table 2). We subsequently deployed integrated localization prediction tools, including protcomp 9.0,^5^ signalP 5.0 (Almagro Armenteros et al., 2019), and secretomeP (Bendtsen et al., 2004). These examinations revealed a total of 580/1,142 extracellular proteins comprising 316 classically secreted (possess the standard Sec/SPI secretory signal peptides) and 264 alternatively secreted proteins (Figure 7A). Additional comparative studies showed the exclusive presence of 23 pathogenesis-related hydrolytic enzymes, including xylogycase (PHYCA_106898), endoglucanase (PHYCA_540248), cutinase (PHYCA_575164), cellulase-2/CNHII (PHYCA_4653), metallopeptidase (PHYCA_13998), peptidase M56 (PHYCA_506653), cysteine-rich secretory proteins/CRISPs (PHYCA_564248), glycosyl hydrolase (PHYCA_115338) among others (Figures 7B,C). These results support our position that PcApt1 likely contributes to the pathogenesis of *P. capsici* through regulating the secretion of essential hydrolytic enzymes and pathogenicity/virulence determinants.

Furthermore, the recovery of 481 proteins comprising of cellulase-A/celA (PHYCA_100188), laccase (PHYCA_64859), catalase (PHYCA_545159), and types necrosis- and ethylene-inducing proteins/NPP1 (PHYCA_576423, PHYCA_9298, PHYCA_129784, PHYCA_544885, and PHYCA_129892) from the extracellular milieu of both the wild-type and Δ*Pcapt1* strains (Figure 7B), suggest the trafficking of these group of proteins out of the cell is likely independent PcApt1 function and hence, partly validate the reasoning that PcApt1 directly or indirectly mediates the selective secretion of proteins and ions during morpho-physiological development of *P. capsici*.

## Discussion

Here, we identified and functionally characterized the ortholog of *S. cerevisiae* Drs2 (PcApt1) in *P. capsici* and found that PcApt1 is involved in the vegetative growth and cell membrane-associated stress response. Moreover, PcApt1 plays a pivotal role in extracellular laccase activity, lipid transport, and export of hydrolytic enzymes and severely truncates the virulence of *P. capsici*. To our knowledge, this is the first report on the contributions of P-type ATPase Drs2 orthologs to the pathophysiological development of oomycetes.

Several lines of evidence demonstrated that APTs/flippases act as regulators of vegetative growth. In *S. cerevisiae*, except for NEO1, the other four flippase genes are not essential for viability. Still, the quadruple mutant (Δ*drs2*Δ*dnf1*Δ*dnf2*Δ*dnf3*) is lethal, suggesting that flippases exist overlapping functions in growth (Hua et al., 2002; Daleke, 2007). In the filamentous fungi *A. nidulans*, targeted disruption of genes coding for *DNFA* and *DNFB* (homolog gene of *DRS2*) reduced radial growth. Also, double gene deletion of *DNFA* and *DNFB or DNFC* and *DNFD* is lethal, indicating that flippases also play a different role in the vegetative growth in *A. nidulans* (Schultzhaus et al., 2015, 2019). In the plant pathogenic fungi *F. graminearum*, except for *FgDNFA*, the other flippases, including *FgDNFB* (homolog of *DRS2*), are not critical for vegetative growth (Yun et al., 2020). Similarly, the loss of two flippase genes *MgPDE1*, and *MgAPT2* (homolog gene of *DRS2*), had no adverse influence on the vegetative growth of *M. grisea.* In yeast, expression of *MgAPT2* in Δ*dnf1*Δ*dnf2*Δ*dnf3* triple mutant can restore the ability to grow in the presence of CFW, but failed to rescue the temperature-sensitive growth defect of the Δ*drs2* strain, indicating that MgApt2 shows partial functions of the Drs2 family of APTs (Balhadere and Talbot, 2001; Gilbert et al., 2006). We demonstrated that the deletion of *PcAPT1* attenuated growth and triggered an approximately 28% reduction in the vegetative growth of Δ*Pcapt1* strains relative to the wild-type strain. These results collectively revealed that Drs2 homologs show similar and distinct roles in various species.

P4-ATPases can transport specific phospholipids from the exoplasmic to the cytoplasmic leaflet of biological membranes to generate and maintain the asymmetrical distribution of aminophospholipids in cell membranes (Tang et al., 1996; Coleman et al., 2009; Zhou and Graham, 2009). In *S. cerevisiae*, removal of Drs2 and Dnf3 disrupts the transport of fluorescently labeled-PS, -PE, and -PC from the exoplasmic to the cytoplasmic leaflet of biological membranes in the post-Golgi secretory vesicles (Tang et al., 1996; Gomes et al., 2000; Alder-Baerens et al., 2006). In addition, deletion of plasma membrane-associated P4 ATPases Dnf1 and Dnf2 contribute to an abnormal exposure of endogenous aminophospholipids at the cell surface (Pomorski et al., 2003; Stevens et al., 2008). These data indicated that P4-ATPases including Drs2, play an indispensable role in phospholipids transport in yeast. Hu and Kronstad found that APT1 (homolog of yeast Drs2) participates in membrane asymmetry. Deletion of *APT1* resulted in the exposure of PE on the outer side of the plasma membrane, suggesting that APT1 is responsible for inward translocation of PE across the plasma membrane in the fungal pathogen *C. neoformans* (Hu and Kronstad, 2010). Previous studies have shown that AnDnfA and AnDnfB play a pivotal role in the asymmetric distribution of phosphatidylserine in the phytopathogenic fungus *A. nidulans* (Schultzhaus et al., 2015). Recently, Yun et al. (2020) found that FgDnfB and FgDnfD are involved in the transport of phosphatidylcholine. Our findings showed that P4 ATPase PcApt1 specifically transports PS rather than PE and PC in *P. capsici*. Taken together, these results showed that P4 ATPases play an indispensable role in the phospholipids transport and individual P4 ATPase showed different substrate specificities. Yeast Drs2 transport fluorescently labeled-PS, -PE, and -PC across the plasma membrane (Tang et al., 1996; Gomes et al., 2000; Alder-Baerens et al., 2006), while Drs2 homologs take part in translocation of different phospholipids in the fungal pathogens (Hu and Kronstad, 2010; Schultzhaus et al., 2015; Yun et al., 2020). In our study, PcApt1 (homolog of Drs2) specifically transports PS, indicating that the role of Drs2 in phospholipids transport likely varies between species. We speculated that other P4 ATPases possibly exist and complement the transport of PE and PC in the plant pathogenic oomycete *P. capsici*. Identifying the additional P4 ATPases in *P. capsici* will be necessary for understanding the function of P4 ATPases in the asymmetric distribution of phospholipids on the biological membrane.

P4 ATPases positively modulate the pathogenesis of multiple fungal pathogens. For instances, studies have shown that two putative APTs (MgApt2 and MgPde1) play an essential role in promoting the virulence of the rice blast fungus; hence, loss of MgPde1 attenuated the development of penetration hypha during plant infection. MgApt2, a Drs2 homolog in *S. cerevisiae*, is required for foliar and root infection of *M. grisea* (Balhadere and Talbot, 2001; Gilbert et al., 2006). Moreover, in the opportunistic fungal pathogen *C. neoformans*, deletion of *APT1* (homolog of yeast Drs2) reduced virulence in a mouse inhalation model of cryptococcosis (Hu and Kronstad, 2010). These findings suggest Drs2 homologs positively regulate the virulence of microbial pathogens. We identified a Drs2 homolog PcApt1 and demonstrated that the targeted gene deletion of *PcAPT1* significantly attenuated virulence of *P. capsici* on leaves and etiolated hypocotyls of Bell pepper, indicating that PcApt1 play an indispensable role in infection of *P. capsici*. Collectively, these data reveal that P4 ATPases are essential for the virulence of various pathogens. Studies have shown that laccase is an important virulence factor between fungi and host interactions (Zhu and Williamson, 2004; Chi et al., 2009). Additionally, deletion of *PlMAPK10*, *PsMPK7*, and *PsHSF1* significantly reduced the extracellular laccase secretion and pathogenicity of the plant pathogenic oomycetes *P. litchii* and *P. sojae*, respectively (Gao et al., 2015; Sheng et al., 2015; Jiang et al., 2018), indicating that laccase possibly associated with the pathogenicity of oomycetes. In our study, loss of *PcAPT1* failed to produce oxidized ABTS based on no dark purple staining around the mycelial mat in compared to the wild-type, suggesting that deletion of *PcAPT1* disrupted extracellular laccase activities in *P. capsici*. Gilbert et al. found that deletion of *MgAPT2* (homolog of yeast *DRS2*) impaired the secretion of a range of extracellular enzymes and infection in the rice blast pathogen *M. grisea* (Gilbert et al., 2006), suggesting that Drs2 homolog participates in the secretion of extracellular enzymes. Therefore, we infer that PcApt1 also possibly mediates the secretion of other extracellular enzymes in *P. capsici*. Identifying the extracellular enzymes associated with PcApt1 will be conducive to understanding the pathogenicity defect of the Δ*Pcapt1* strains.

During host invasion and pathogenic differentiations, pathogenic microbes secrete a wide array of hydrolytic enzymes, including metallopeptidases, laccases, endoglucanases, and Cellobiohydrolases cellulases, hemicellulases, pectinases, cutinases, xylanases, xyloglucanases, lipase, among others (Gravi et al., 2012; Singh et al., 2019). These enzymes mediated the degradation of defense-related proteins secreted from the host in the extracellular matrix, subvert cell wall, membrane integrity of the host cell. Hydrolytic enzymes suppress the host’s resistance against the invading pathogen. For instance, the secretion of metallopeptidase promotes the virulence of *Candida albicans* and *Cryptococcus neoformans* by mediating the degradation of collagen and fibronectin (Rodier, 1999; Rodier et al., 2001). Also, in *Botrytis cinerea*, the secretion of xyloglucanase triggered cell death (necrosis) in the host and enhanced the progressive infection by *B. cinerea* (Zhu et al., 2017). Here, we demonstrated that targeted gene disruption of *PcAPT1* in the hemibiotrophic phytopathogenic oomycetes *P. capsici* impaired the secretion of pathogenesis-associated hydrolytic enzymes and severely compromised the virulence of the defective strains against susceptible pepper seedlings as either a direct or indirect consequence of *PcAPT1* dysfunction.

In summary, we demonstrated that PcApt1, a member of the P4-ATPases family, exerts a profound influence on the physiological, pathogenic, and infectious development of *P. capsici* through direct or indirect modulation of classical and non-classical secretion of hydrolytic enzymes and possibly other growth and virulence promoting factors. These results also project PcApt1 as a potent and durable target for developing disease control strategies.

## Data Availability Statement

The original contributions presented in the study are included in the article/Supplementary Material, further inquiries can be directed to the corresponding author/s.

## Author Contributions

QC conceived the study. QC and JN designed the experiments and revised the manuscript. CY, BZ, RW, HC, PL, and BL performed the experiments. JN and CY analyzed the data. CY drafted the manuscript. All authors contributed to the final manuscript.

## Conflict of Interest

The authors declare that the research was conducted in the absence of any commercial or financial relationships that could be construed as a potential conflict of interest.

## Publisher’s Note

All claims expressed in this article are solely those of the authors and do not necessarily represent those of their affiliated organizations, or those of the publisher, the editors and the reviewers. Any product that may be evaluated in this article, or claim that may be made by its manufacturer, is not guaranteed or endorsed by the publisher.

## Footnotes

1
https://fungidb.org/fungidb/app

2
http://pfam.xfam.org/search

3
http://grna.ctegd.uga.edu/

4
https://mycocosm.jgi.doe.gov/Phyca11/Phyca11.home.html

5
www.softberry.com
