# Supplementary material for: A Putative P-Type ATPase Regulates the Secretion of Hydrolytic Enzymes, Phospholipid Transport, Morphogenesis, and Pathogenesis in Phytophthora capsici
Source: Front Plant Sci. 2022 May 10;13:852500. doi: 10.3389/fpls.2022.852500 (PMC9127794; doi:10.3389/fpls.2022.852500)
Supplement: Supplementary file 1 [file Data_Sheet_1.docx]

**Supplementary material**


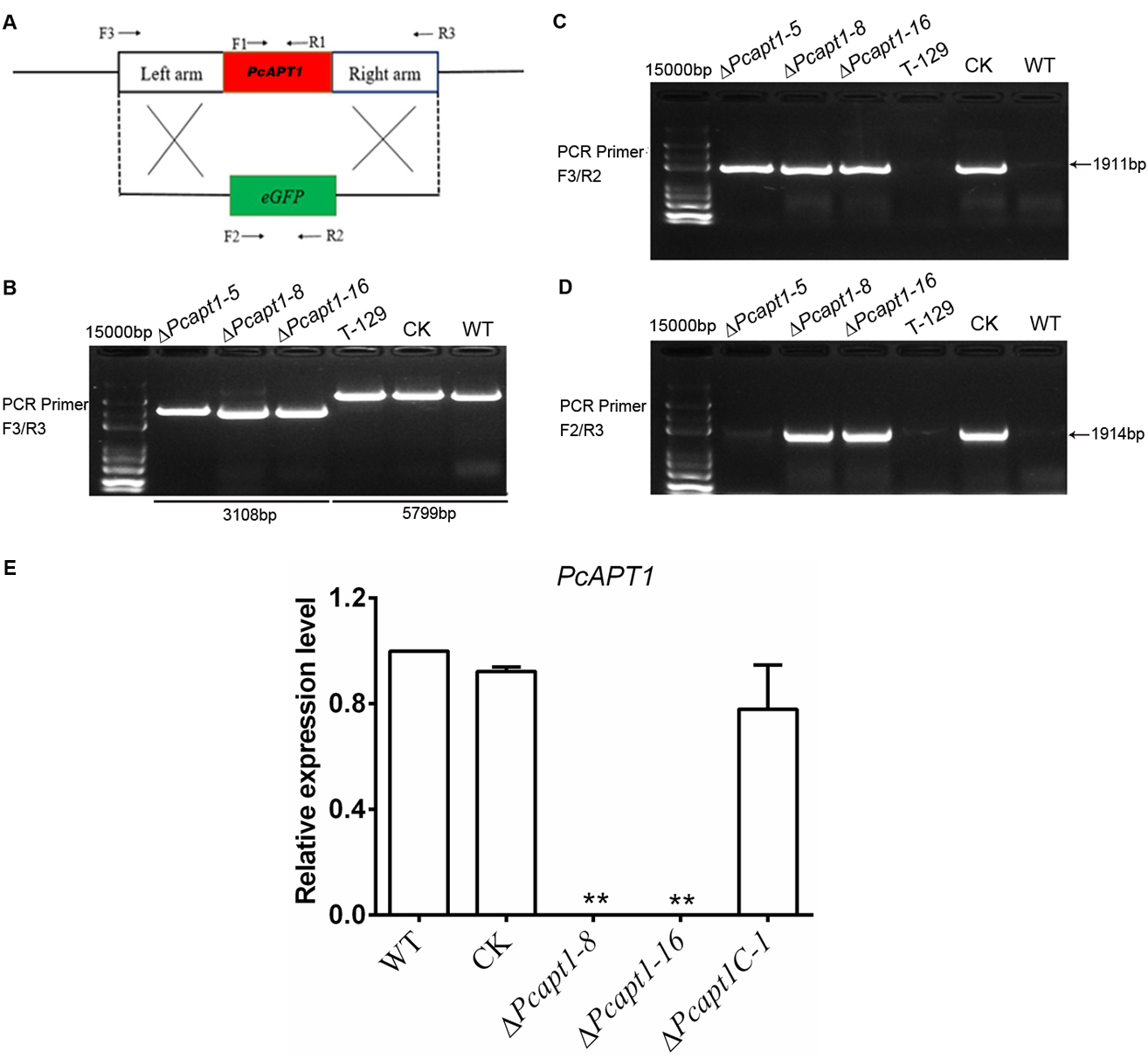


**Figure S1. CRISPR/Cas9-mediated *PcAPT1* knock out.**

**(A)** Strategy of homology-directed repair (HDR)-mediated replacement of the *PcAPT1* open reading frame (ORF) with an eGFP ORF.

**(B)-(D)** Identification of the *PcAPT1* deletion mutants by the related primer pairs. F3/R3 primer pairs were used to amplify the full length from left arm to right arm with a 3.108 kb band in the ∆*Pcapt1* strains. F3/R2 and F2/R3 primer pairs were used to identify the ORF of *PcAPT1* was replaced with eGFP.

**(E)** Relative expression levels of the *PcAPT1* gene in the ∆*Pcapt1* strains were analyzed by qRT-PCR with β-tubulin as endogenous reference gene. Statistical differences were calculated by multiple t-tests using GraphPad Prism at p ≤ 0.01.


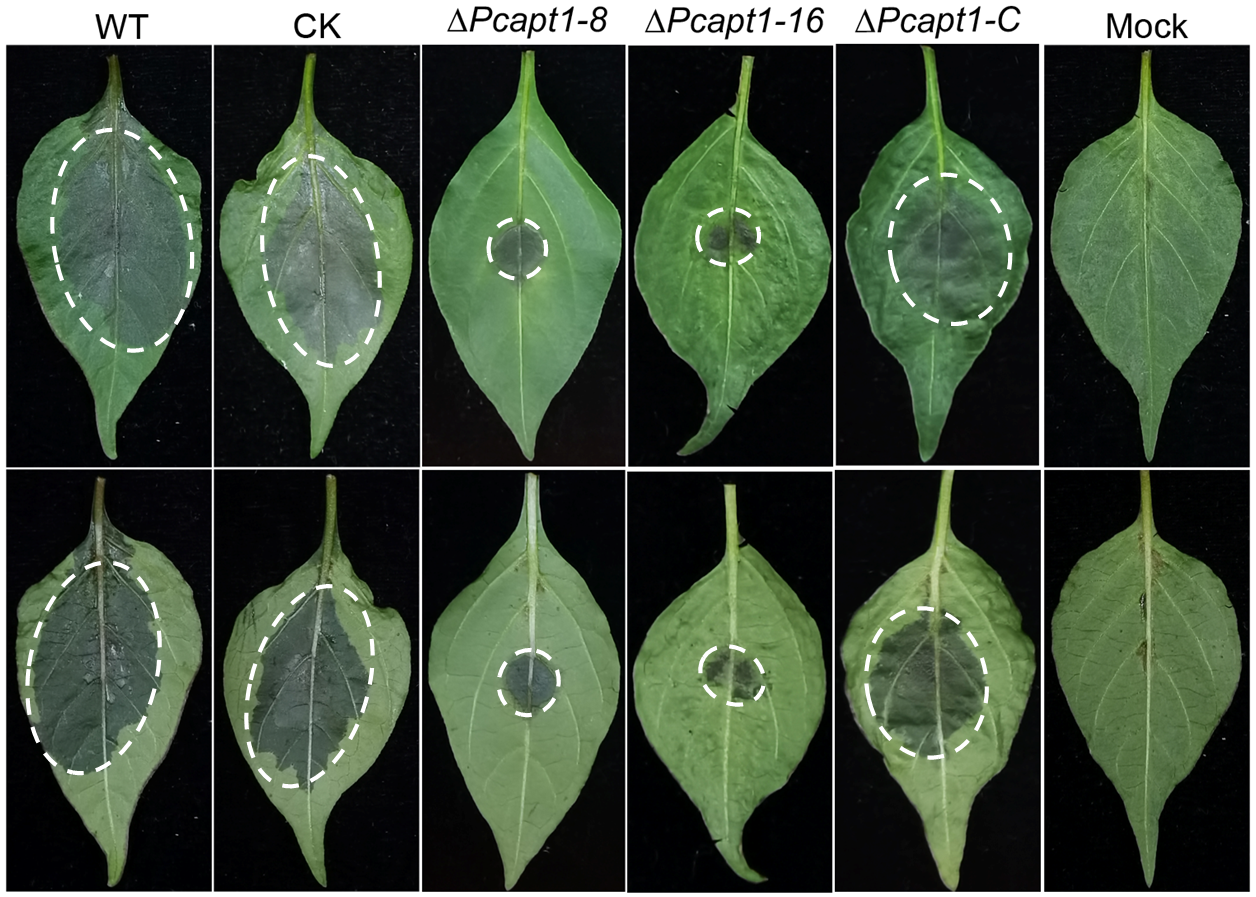


**Figure S2. Pathogenicity of zoospores from the ∆*Pcapt1* strains on pepper leaves.**

Infection of the ∆*Pcapt1* strains to detached Bell pepper leaves was significantly decreased. Bell pepper leaves were inoculated with zoospores of WT, complemented, CK, and the ∆*Pcapt1* strains. Photographs were taken at 3 days post-inoculation (dpi).
