# Supplementary material for: A Putative P-Type ATPase Regulates the Secretion of Hydrolytic Enzymes, Phospholipid Transport, Morphogenesis, and Pathogenesis in Phytophthora capsici
Source: Front Plant Sci. 2022 May 10;13:852500. doi: 10.3389/fpls.2022.852500 (PMC9127794; doi:10.3389/fpls.2022.852500)
Supplement: Supplementary file 2 [file Table_1.docx]

**Table S1** List of primer pairs used in this study

| Primers | Sequences（5’ → 3’） | Function |
| --- | --- | --- |
| *sgPcAPT1-*1F | CTAGCGCCGTGCTGATGAGTCCGTGAGGACGAAACGAGTAAGCTCGTCCACGGCGTGTCGTACGGCCG | *PcAPT1-*sgRNA1-Cas9 expression plasmid |
| *sgPcAPT1-*1R | AAACCGGCCGTACGACACGCCGTGGACGAGCTTACTCGTTTCGTCCTCACGGACTCATCAGCACGGCG |  |
| *sgPcAPT1-*2F | CTAGCCCAAGCCTGATGAGTCCGTGAGGACGAAACGAGTAAGCTCGTCGCTTGGAGATTTGTCTCGAG | *PcAPT1-*sgRNA2-Cas9 expression plasmid |
| *sgPcAPT1-*2R | AAACCTCGAGACAAATCTCCAAGCGACGAGCTTACTCGTTTCGTCCTCACGGACTCATCAGGCTTGGG |  |
| *pBS-PcAPT1-*LF | GGGCCCCCCCTCGAGGTCGACG GTATAAGACGTGTGTGGCTTCTG | upstream sequence of *PcAPT1* for gene replacement plasmid |
| *pBS-PcAPT1-*LR | TGCTCACCATCGTGGACGATCCCAGACC |  |
| *pBS-eGFP-*F | ATCGTCCACGATGGTGAGCAAGGGCGAG | Donor DNA for gene replacement plasmid |
| *pBS-eGFP-*R | GTCGATGAAACTTGTACAGCTCGTCCATGC |  |
| *pBS-PcAPT1-*RF | GCTGTACAAGTTTCATCGACGCGGAAGTCAAAAC | downstream sequence of *PcAPT1* for gene replacement plasmid |
| *pBS-PcAPT1-*RR | CGGCCGCTCTAGAACTAGTGGCCCGTATCGACCTGCCA |  |
| F1 | CAACTTTTACTTCCTGCTGGT | Identification of *PcAPT1* mutants |
| R1 | TCCAGAGAAGTTATTGACGTCGT |  |
| F2 | ATGGTGAGCAAGGGCGAGGA | Identification of *PcAPT1* mutants |
| R2 | TCGGCATGGACGAGCTGTACAAG |  |
| F3 | ACAGTGAGCATCGACATTGACAA | Identification of *PcAPT1* mutants |
| R3 | AGATTTTCACCTGCAAGCTACGGT |  |
| PF | CGACGGTATCGATGATATCGGAGGCTCAGGCTCTAAACT | Complementation of *PcAPT1* mutants |
| PR | CGCCCTTGCTCACCATGGTGTTCCTGCAAAATGTGGTAGT |  |
| *PcAPT1-QF* | GAAGTAGCCATCACAGTC | qRT-PCR analysis for *PcAPT1* gene |
| *PcAPT1-QR* | CGTGTCTATCATCGTCTG |  |
